# Towards the Objective Identification of the Presence of Pain Based on Electroencephalography Signals’ Analysis: A Proof-of-Concept

**DOI:** 10.3390/s22166272

**Published:** 2022-08-20

**Authors:** Colince Meli Segning, Jessica Harvey, Hassan Ezzaidi, Karen Barros Parron Fernandes, Rubens A. da Silva, Suzy Ngomo

**Affiliations:** 1 Department of Applied Sciences, Université du Québec à Chicoutimi (UQAC), Saguenay, QC G7H 2B1, Canada; 2 Laboratoire de Recherche Biomécanique et Neurophysiologique en Réadaptation Neuro-Musculo-Squelettique (*Lab BioNR*), Université du Québec à Chicoutimi (UQAC), Saguenay, QC G7H 2B1, Canada; 3 Remix Health Clinic, Chicoutimi, QC G7H 7K9, Canada; 4 Department of Health Sciences, Université du Québec à Chicoutimi (UQAC), Saguenay, QC G7H 2B1, Canada; 5 School of Medicine, Pontifical Catholic University of Parana (PUCPR), 485-Hipica, Londrina 86072-360, PR, Brazil; 6 Centre Intégré de Santé et Services Sociaux du Saguenay-Lac-Saint-Jean (CIUSSS SLSJ), Specialized Geriatrics, Services-Hôpital de La Baie, Saguenay, QC G7H 7K9, Canada

**Keywords:** pain, EEG signal, coefficient of variation of the upper envelope (CVUE), beta EEG frequency band

## Abstract

This proof-of-concept study explores the potential of developing objective pain identification based on the analysis of electroencephalography (EEG) signals. Data were collected from participants living with chronic fibromyalgia pain (*n* = 4) and from healthy volunteers (*n* = 7) submitted to experimental pain by the application of capsaicin cream (1%) on the right upper trapezius. This data collection was conducted in two parts: (1) baseline measures including pain intensity and EEG signals, with the participant at rest; (2) active measures collected under the execution of a visuo-motor task, including EEG signals and the task performance index. The main measure for the objective identification of the presence of pain was the coefficient of variation of the upper envelope (CVUE) of the EEG signal from left fronto-central (FC5) and left temporal (T7) electrodes, in alpha (8–12 Hz), beta (12–30 Hz) and gamma (30–43 Hz) frequency bands. The task performance index was also calculated. CVUE (%) was compared between groups: those with chronic fibromyalgia pain, healthy volunteers with “No pain” and healthy volunteers with experimentally-induced pain. The identification of the presence of pain was determined by an increased CVUE in beta (CVUE_β_) from the EEG signals captured at the left FC5 electrode. More specifically, CVUE_β_ increased up to 20% in the pain condition at rest. In addition, no correlation was found between CVUE_β_ and pain intensity or the task performance index. These results support the objective identification of the presence of pain based on the quantification of the coefficient of variation of the upper envelope of the EEG signal.

## 1. Introduction

Chronic pain is defined as pain lasting or recurring for longer than three months, despite treatment [1]. The prevalence of moderate to severe chronic pain has been reported in 20% to 30% of the adult population [2,3,4]. Chronic pain is often accompanied by functional impairment and decreased physical capacity and has recently been reported by the Global Burden of Disease reviews to be one of the most prominent causes of disability, worldwide [2,5,6,7].

It is well-established that chronic pain is related to the alteration of the modulation of the central nervous system, and, specifically, in terms of the dysfunction of cortical motor regions [8,9]. Nowadays, pain assessment is often associated with self-reporting of the presence/intensity of pain from a subjective approach, based on the individual’s lived experience of pain [10,11,12]. However, this indirect approach could fail to determine pain in individuals who are unable to verbally express it, such as infants, or patients with cognitive disorders, for example [13,14]. In this sense, the objective assessment of pain from neurophysiological measurements should certainly be useful.

Brain imagery techniques, such as functional magnetic resonance imaging (fMRI) or electroencephalography (EEG) are helpful to explore how the brain works. These techniques can help identify the brain’s functional pattern related to the pain process [15,16,17,18,19]. For instance, Nir et al. [20] carried out the detection of pain with experimentally-induced pain through a thermal contact-heat simulator in 18 healthy volunteers, in a resting state. Results showed that the peak of alpha frequency calculated from EEG signals could be used as an objective measure to detect pain perception. Later, Nir et al. [21] moved this research forward to focus on exploring the power spectral density in alpha-1 (8–10 Hz). Results suggested that the power spectral density of alpha-1 could serve as a direct and stable measure of pain perception. More recently, Hu and Iannetti, [22] found that gamma-band (60–85 Hz) event-related synchronization (γ-ERS) from EEG signals predict pain perception across humans and rodents. Another study by May et al. [23] investigated the neurophysiological representation of pain intensity in 31 chronic back patients. Their results show a significant positive association between ongoing pain intensity and the amplitude of beta (14–29 Hz) and gamma oscillations. All these studies suggest a more objective method of predicting the presence of pain.

To the best of our knowledge, most EEG studies used the frequency-domain analysis approach based on the Fast Fourier Transform (FFT) method, which does not consider the non-linearity of cortical activity. Brain oscillation variability, across and within individuals, poses major activity analysis and interpretation challenges [24], and even more in the context of pain, which does not appear as an anatomical structure in the brain, but rather as a dysfunction of cerebral excitability, dispersed in cerebral oscillations. Thus, the non-linearity and time varying of cortical signals increase the difficulty in identifying a concrete or a specific biomarker of the presence of pain.

To surround the non-linearity of the EEG signal emitted by brain activity, a few studies have investigated time-frequency analysis methods. In particular, Cole and Voytek [25] highlighted the stability of the EEG signal envelope, despite the high variability of the overall neural signal, suggesting that envelope analysis is more appropriate for better features’ detection. Analysis based on the envelope signal offers more stable measures, providing a practical description of neural dynamics [26,27]; the envelope can then be used to accurately describe EEG signal amplitude changes over time. The envelope of the EEG signal can be thought of as the curve encompassing the upper and lower extremes of this signal. The upper envelope represents the maxima points of the curve, interconnected using a cubic spline interpolation, whereas the lower envelope is the minima points, interconnected using a cubic spline interpolation [28]. The coefficient of variation of the signal envelope constitutes a scale-independent measure to quantify cortical activity, making it suitable for comparisons across different EEG time series [29]. To address the non-linearity as well as the inter- and intra-subject variability of brain activity, we proposed to explore the coefficient of variation of the upper envelope (CVUE) of the EEG signal as a scale-independent descriptor of signal morphology [26,27]. Therefore, the present proof-of-concept study aimed to explore the potential of developing objective pain identification (with pain or no pain) based on the CVUE analysis method in individuals with chronic pain. 

## 2. Materials and Methods

### 2.1. Participants

From a cross-sectional experimental design, two groups of individuals participated in this study: (1) Group 1 included individuals suffering from chronic pain, such as fibromyalgia (FM), designed as the case group and (2) Group 2 related to healthy volunteers as a control who participated in two main parts of the experiment: with “No pain” and with experimental tonic pain, designed as the control group.

Fibromyalgia is a chronic condition, mainly reported as widespread musculoskeletal chronic pain, with a negative effect on physical functions; and in particular a severe limitation of movements of the neck–shoulder region [8,9]. The inclusion criteria for fibromyalgia subjects were: (1) chronic fibromyalgia pain (CFP), i.e., pain for at least three months [1], meeting the American College of Rheumatology criteria, including Widespread Pain Index (WPI) [30] and (2) right-handed, based on the Edinburgh Handedness questionnaire. 

For the second group, “Healthy volunteers” were defined as participants without self-reported evidence or clinical symptoms and signs of heart and lung disease. These participants were aged at least 18 years old, and right-handed, based on the Edinburgh Handedness questionnaire [31,32], and had not presented any pain in the last seven days prior to the experiment or any musculoskeletal disorders affecting the dominant upper limb. For both groups, chronic fibromyalgia pain or CFP and healthy volunteers, the exclusion criteria were to be on antidepressant medication or to present any history of neurological or psychiatric problems. Throughout the study, participants maintained their regular activities and medication. 

Participants were subjected to a single experimental session. One trained evaluator performed all the measurements. The local Research Ethics Committee (CER #602.442.04) approved the study and all participants gave written consent for their participation.

Table 1 reports participants’ characteristics for both groups. Four (4) right-handed women with mean age: 42.75 years, range: 24–61 years, pain duration range: 2–43 years were classified in the chronic fibromyalgia pain group, while 7 right-handed participants (1 woman, mean age: 36.83 years, range: 24–45 years) were classified in the healthy volunteers’ group (see Table 1).

Pain intensity was assessed using a 0–10 verbal numerical rating scale (NRS), where 0 corresponds to no pain and 10 to the worst imaginable pain [33]. Widespread Pain Index (WPI) is based on the number of body pain sites or trigger points and is a fibromyalgia specific index [34].

### 2.2. Procedure

The experimental design was comprised of two main parts: (1) Baseline measures (at rest for 60 s, prior to beginning the visuo-motor task for all participants, including participants with CFP, participants with induced pain (Healthy with induced pain (HWIP)) and participants without induced pain (Healthy with “No pain” (HWNp)); (2) the active measures during visuo-motor task for 4 min for all participants including CFP, HWNp and HWIP.

#### 2.2.1. Experimental Pain

Pain was induced in the healthy volunteer group with a topical application of a ~1 cm wide band of capsaicin cream (1%) (~1 mm thick) on the right upper trapezius. The plateau of pain was attained about 30 min following the application [35]. The plateau corresponds to the moment when the participant consecutively gives the same intensity 3 times (pain intensity was assessed every 2 min); capsaicin induces moderate pain intensity (3–5/10 on a numerical rating scale or NRS) [36].

#### 2.2.2. Baseline Measures, Participant at Rest

**Figure 1:**
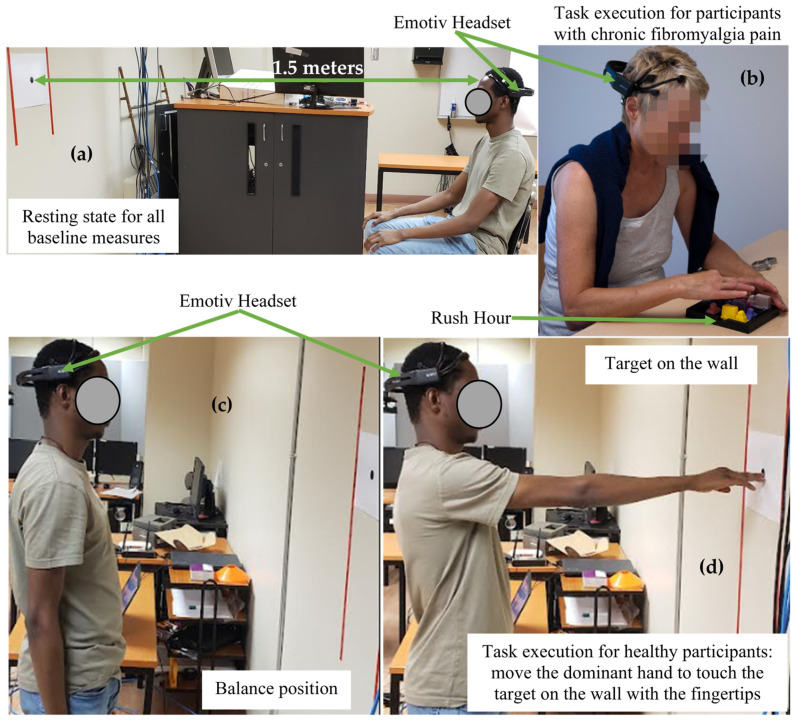
Experimental conditions: (**a**)—resting state for baseline measures, (**b**)—task execution for participants with chronic fibromyalgia pain, (**c**)—balance position during task execution for healthy participants and (**d**)—task execution for healthy participants without pain and during the plateau of induced pain.

Pain intensity and EEG signals were recorded for all participants, at rest. Pain intensity was measured using NRS. EEG signals were recorded for 60 s with the participant comfortably seated on an adjustable chair at 1.5 metres with eyes opened focused on a black point on the wall (Figure 1a). For the healthy group in the induced pain condition, the last 60 s during the plateau of the experimental pain were considered for EEG baseline measures.

#### 2.2.3. Active Measures, Participant Executing a Visuo-Motor Task

For all participants, EEG signals were recorded during task execution.

#### 2.2.4. Task for Chronic Fibromyalgia Pain Group

The visuo-motor task was performed during 4 min in a sitting position to try to limit the pain induced by the task to the upper limbs, knowing that fibromyalgia is characterized by widespread bodily pain. The task was to extract a target car from a 3D puzzle placed on a table at a distance from the right forearm (elbow at the edge of the table), among several other cars blocking the exit, in a rush hour context (Figure 1b). Indeed, “Rush Hour” was the name of the game [37]. Moving the car in a limited time, as quickly as possible, requires moving the upper limbs, knowing that this part of the body is painful and disabled in persons living with chronic fibromyalgia pain. One cycle of the task called to move the target car until they were able to extract it from the blocked circulation. 

#### 2.2.5. Task Performance in the Chronic Fibromyalgia Pain Group

One cycle of the task consisted of moving the target car until its extraction from blocked circulation. The task performance index was calculated as a ratio of the number of cycles performed to the total time allocated for the task (4 min). A high score of task performance index means high performance; a perfect performance would give a ratio of 1. 

#### 2.2.6. Task for the Healthy Group

The task required to touch a target on the wall with the fingertips of the right hand for 4 min. The target was set at eye level, the participant in a standing posture, according to the height of each participant and at a distance equal to that of their outstretched upper limb (Figure 1d). This task was executed without and with pain (during the plateau of pain). One cycle of the task required participants to move the dominant hand to touch the target on the wall with the fingertips, then return to the balance position (arms held vertically along the body (Figure 1c)).

#### 2.2.7. Task Performance for the Healthy Group

Task performance index was assessed as a ratio of the number of cycles to the total time allocated for the task (4 min or 240 s). A high score of task performance index reflects high performance; a perfect performance would give a ratio of 1.

### 2.3. EEG Data Acquisition

**Figure 2:**
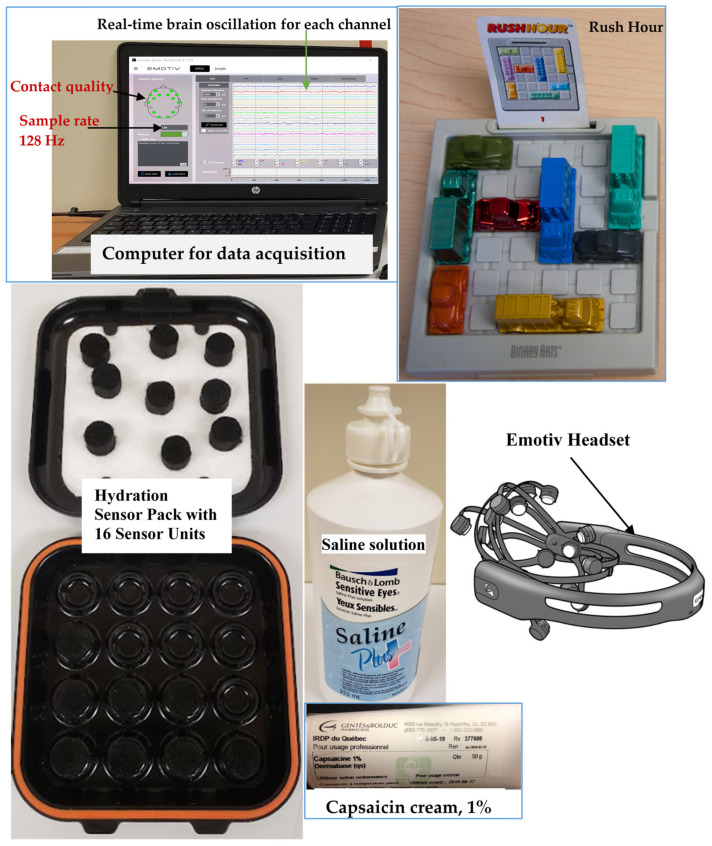
Equipment for data collection.

All participants were instructed on how to perform their visuo-motor task once before recording EEG signals. The acquisition of EEG data was conducted first at baseline (resting measures) during 60 s in a sitting position in fibromyalgia and heathy participants (the first 60 s without pain and the first 60 s during the plateau of pain in experimental pain). Figure 2 presents the equipment for data collection. At baseline measures, participants were instructed to remain as still as possible and to focus their sight on the black point on the wall, to minimize muscular artifacts. Healthy participants after baseline EEG measures were invited to perform the task in an upright position during 4 min or 240 s (active measures), while the chronic pain group performed it in a sitting position. According to the stabilization time of the experimentally-induced pain by the capsaicin (30 min), EEG signals were recorded for 30 min in the healthy group, during which pain intensity was collected every 2 min. Only the first 4 min during the plateau of pain were considered for actives measures.

A wireless EEG device was used, with an Emotiv EPOC+ 16-channel headset (Emotiv Systems Inc., San Francisco, CA, USA), using the international 10–20 system and including 14 active and 2 ground electrodes. Impedance was maintained in a 10–20 KΩ range, using saline liquid. EEG data were acquired with an internal sampling frequency of 2048 Hz. Data were then digitalized using the embedded 16-bit analog-to-digital converter and downsampled to 128 samples per second before being transmitted to the acquisition computer. The digitalized EEG signals were online filtered by the EPOC+ hardware with a 5th-order digital sinc filter using bandpass of 0.2–45 Hz and notch digital filter at 60 Hz (for North America) to eliminate line frequency noise.

#### 2.3.1. EEG Data Preprocessing Analysis

Electrodes retained for EEG signal analysis were FC5 and T7 since these are the only electrodes (bilaterally) that cover the recommended positioning of the premotor cortex, supplementary motor areas, and the primary motor cortex, according to the EPOC+ headset used in this study [38]. Moreover, previous studies reported the involvement of the motor cortex areas in the nociception process [39,40]. Because all participants were right-handed, the left FC5 and T7 were selected.

EEG data from selected electrodes (FC5 and T7) were preprocessed using MATLAB software analysis (The MathWorks Inc., Natick, MA, USA). To remove noise from the EEG signals, the direct current (DC) voltage offset, i.e., an offsetting of a signal from 0, was firstly removed using the simplest method consisting of subtracting the average value (approximatively 4200 µV) from the entire selected data channel. The second step was to remove the rest of artifacts due to eye blinks or eye movements and electromyography (EMG) signal, all well-known noise such as yawning, coughing, etc., as well as the poor electrode contact quality identified during the experiments. An outlier detection and replacement filter was used to remove noise in the second step [41,42]. To this end, the outlier’s values were defined as EEG values that were over 1.5 interquartile ranges above the upper quartile (75 percent) or below the lower quartile (25 percent) and detected using quartiles find method. Then, the linear interpolation of neighboring, non-outlier values method was used to replace the detected outlier’s values.

#### 2.3.2. EEG Frequency Sub-Bands Retained for Signal Analysis

The third step was the selection of the EEG frequency sub-bands for objective analysis including alpha (8–12 Hz), beta (12–30 Hz) and gamma (30–43 Hz) over left fronto-central (FC5) and left temporal (T7) electrodes for further analyses [15,20,21,43,44]. These bands were selected for their involvement in pain neural processing [16,21,44]. Finally, a 5th-order IIR Butterworth band pass filter was used for all selected frequency bands [45].

#### 2.3.3. EEG Data Normalization

The fourth step was to adjust for the inter-variability of EEG data within each frequency band by scaling the EEG data using min–max normalization. This fourth step consists of placing the EEG data in the interval between 0 and 1 [46,47]. Min–max normalization performs a linear transformation on the original data values while preserving relationships among them. The equation formula to achieve this processing was [48,49]

(1) $${\mathrm{EEG}}_{i}^{min\_max}=\frac{{\mathrm{EEG}}_{i}-\min\left(\mathrm{EEG}\right)}{\max\left(\mathrm{EEG}\right)-\min\left(\mathrm{EEG}\right)}$$

where ${\mathrm{EEG}}_{i}$ and ${\mathrm{EEG}}_{i}^{min\_max}$ correspond to the original and min–max normalized EEG values i, respectively, min(EEG) and max(EEG) separately denote the minimum and maximum of the whole EEG signals within the considered frequency band. Finally, the last step was unity normalization, which re-scales the previous min–max normalization values by the weight of each single min–max normalized EEG value. It divides each min–max normalized EEG value by a selected reference. The equation is

(2) $${\mathrm{EEG}}_{fi}^{N}=\frac{{\mathrm{EEG}}_{i}^{min\_max}}{M_{Rf}}$$

where ${\mathrm{EEG}}_{fi}^{N}$ represents the normalized single of min–max EEG values i for frequency band f and $M_{Rf}$ is the reference, defined as the mean of min–max EEG values in the reference interval for frequency band f. For EEG signals at rest, $M_{Rf}$ was taken as the first second of EEG collection for each participant, while in active EEG normalization, $M_{Rf}$ was the 60 s of baseline EEG recordings. The next section was the extraction of neurophysiological parameters in the 1 s with 50% overlapping sliding windows.

#### 2.3.4. Calculation of the Coefficient of Variation of Upper Envelope (CVUE)

The upper envelope of the EEG signal was analyzed in the present study, as it provides information on the morphology of brain signals, as previously proposed by Diaz et al. [26,27]. EEG signals are collected in time domain and generally have non-sinusoidal morphology and are non-stationary since their frequency content varies over time [50,51]. Therefore, the Fast Fourier Transform (FFT) techniques mostly used for measuring EEG signals are not necessary for the more adapted approach. The principle of FFT is to decompose the signal into a sum of sinusoids. Thus, FFT does not consider the dynamics of non-linear aspects that underlie EEG signal generation processes. This explains the importance of analyzing the waveform of brain oscillations, as recently pointed out by Cole and Voytek [25]. Because of the high variability in brain activity, and even more in a context of pain, we chose to calculate the coefficient of variation of upper envelope (CVUE) as a scale-independent descriptor of signal morphology [52] to identify the neurophysiological signature of pain in brain oscillations. The coefficient of variation represents the ratio of the standard deviation to the mean, and it is a useful statistic to compare the degree of variation from one data series to another. The computation of CVUE was performed in alpha (CVUE_α_), beta (CVUE_β_) and gamma (CVUE_ɣ_) both in baseline (at rest) and active (during task) conditions in two steps.

(1) The first step was the calculation of the amplitude of upper envelope (AUE) of EEG signals for frequency sub-bands ($S_{f}={\;S}_{\alpha }={\;S}_{\beta }=S_{ɣ}$), defined as the absolute value of the Hilbert transform, as follows

(3) $$\mathrm{AUE}=\left|\sqrt{S_{f}^{2}+ℋ\left(S_{f}^{2}\right)}\right|$$

where f represents alpha, beta or gamma frequency band and ℋ the Hilbert transform. Advantages of using the Hilbert transform:

The EEG signal is mainly decomposed into narrow frequency sub-bands to obtain the brain activities in the alpha (8–12 Hz), beta (12–30 Hz) and gamma (30–43 Hz) bands, as described above. The variations in the amplitude (envelope) and frequency (fine structure of the signal) at the output of each band are supposed to characterize the activities and different rhythmic aspects of the brain. To extract the envelope without losing part of the information on brain activity contained in the EEG signal, the absolute value (modulus) of the analytical signal was calculated. This analytical signal was obtained by applying the Hilbert transform to the original real-valued EEG signal. The conventional method, based on the Fourier transform, is responsible for the loss of part of the information, due to the complex character of the EEG signal. On the other hand, the quasi-totality of the information is retained if the real-valued signal is expressed in a complex form. Thus, in this context, the use of an analytical (complex) signal by Hilbert transform, which mathematically represents the complexity of the brain activity, is much more relevant [28]. In addition, it is easy to implement and in frequency domain, the Hilbert transform can be seen as an operator that shifts the phase angle of all components of the signal by ±π/2 according to the sign.

AUE of a given cortical oscillation reflects the range of energy over time [53]. The amplitude of upper envelope (AUE) is high when energy is high. Because of the time-variant behaviour and non-linearity of EEG signals, they were segmented in sliding window (epochs) of 1-s duration. AUE of EEG signals was then calculated within each epoch.

(2) The second step was the assessment of the coefficient of variation of upper amplitude in alpha (CVUE_α_), beta (CVUE_β_) and gamma (CVUE_ɣ_) EEG frequency bands. To this end, the mean and standard deviation (std) of AUE were computed in each epoch to obtain CVUE_α_, CVUE_β_ and CVUE_ɣ_ as follows

(4) $${\mathrm{CVUE}}_{f}\left(\%\right)=\frac{\mathrm{std}\left(\mathrm{AUE}\right)}{\mathrm{mean}\left(\mathrm{AUE}\right)}\times 100$$

where f represents the alpha, beta or gamma frequency band. 

The envelope of a signal in a given frequency band can be viewed as a representation of the instantaneous energy in this band [54]. The coefficient of variation provides how much the upper envelope varies over time. In that context, the coefficient of variation provides a scale-independent measure of neuronal activity; it is a measure of the dispersion of the data around the mean (Equation (4)). Therefore, it is a useful parameter to compare the degree of variation from one data series to another, in different conditions beyond the high inter–intra-subject variability that characterizes EEG signals. The intrinsic rhythmic nature of pain depends on the clinical situation, the environment, the moment or the period of the day. Several pain studies use the coefficient of variation to describe pain variation over time [55,56,57]. Low CVUE values reflect more stable sinusoidal oscillation, i.e., more neuronal synchronization or inhibition [54]. In contrast, high CVUE corresponds to neuronal desynchronization [26,54], i.e., more facilitation or disinhibition.

### 2.4. Statistical Analysis

Due to our smaller sample size, a non-normal distribution was observed (validated by the Shapiro–Wilk test), and the comparisons of the coefficient of variation of the upper envelope (CVUE) between groups (chronic pain × healthy with induced pain × healthy with “No pain”) were performed using a non-parametric permutation test with 5000 permutations [58]. Finally, Spearman’s correlation was used to evaluate the relationships between pain intensity and CVUE as well as task performance index. Significance level was set at *p* < 0.01 and the statistical analyses were conducted with the MATLAB version 2018b (The MathWorks Inc., Natick, MA, USA) and SPSS version 24 (IBM Corp., Armonk, NY, USA).

## 3. Results

### 3.1. Pain Intensity

The average of pain intensity was 4.75/10 for participants with chronic fibromyalgia pain and 4/10 for healthy participants at the plateau of induced pain. All measurements were taken with participants at rest; pain intensity was not collected during task execution.

### 3.2. Task Performance

The average of task performance was 0.02/1 for participants with chronic fibromyalgia pain, 0.59/1 for healthy participants at the plateau of induced pain and 0.56/1 for healthy participants with “No pain”. These results suggest that chronic fibromyalgia pain seems to have a more negative impact on functional performance.

### 3.3. EEG Baseline Measures: Coefficient of Variation of Upper Envelope from Alpha (CVUE_α_), Beta (CVUE_β_) and Gamma (CVUE_γ_) EEG Frequency Bands, at Rest

#### 3.3.1. In Participants with Chronic Pain vs. Healthy with “No Pain” (HWNp) at Rest

**Figure 3:**
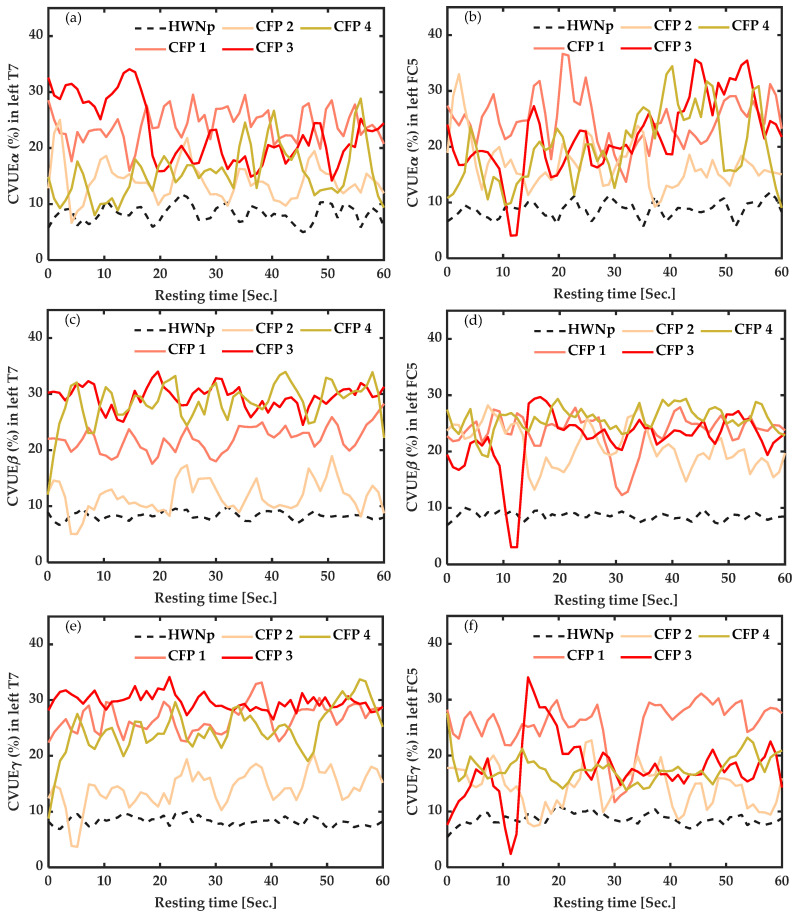
Baseline measures of the coefficient of variation of upper envelope (CVUE) in alpha (CVUE_α_), beta (CVUE_β_) and gamma (CVUE_γ_), at rest. (**a**)—CVUE_α_ from left temporal (T7) data, (**b**)—CVUE_α_ from left fronto-central (FC5) data, (**c**)—CVUE_β_ from left temporal (T7) data, (**d**)—CVUE_β_ from left fronto-central (FC5) data, (**e**)—CVUE_γ_ from left temporal (T7) data, (**f**)—CVUE_γ_ from left fronto-central (FC5) data. The black dotted line indicates the average of CVUE of healthy with “No pain” (HWNp). The colored lines represent CVUE of the four participants with chronic fibromyalgia pain (CFP_1-4_). The graphs were smoothed by a 5th-order of Savitzky–Golay filter. CVUE_β_ from FC5 data was significantly increased in all CFP.

As illustrated in Figure 3, at rest, CVUE values of all participants with chronic fibromyalgia pain were higher than 10%, while the average CVUE of seven healthy participants with “No pain” (HWNp) was less than 10%. The difference was significant in all frequency bands but more marked in CVUE_β_ from FC5 (Figure 3d) (*p* < 0.001), reflecting neuronal desynchronization (more disinhibition) of the motor cortex area.

#### 3.3.2. In Healthy Participants with Induced Pain (HWIP) vs. Healthy with “No Pain” (HWNp) at Rest

**Figure 4:**
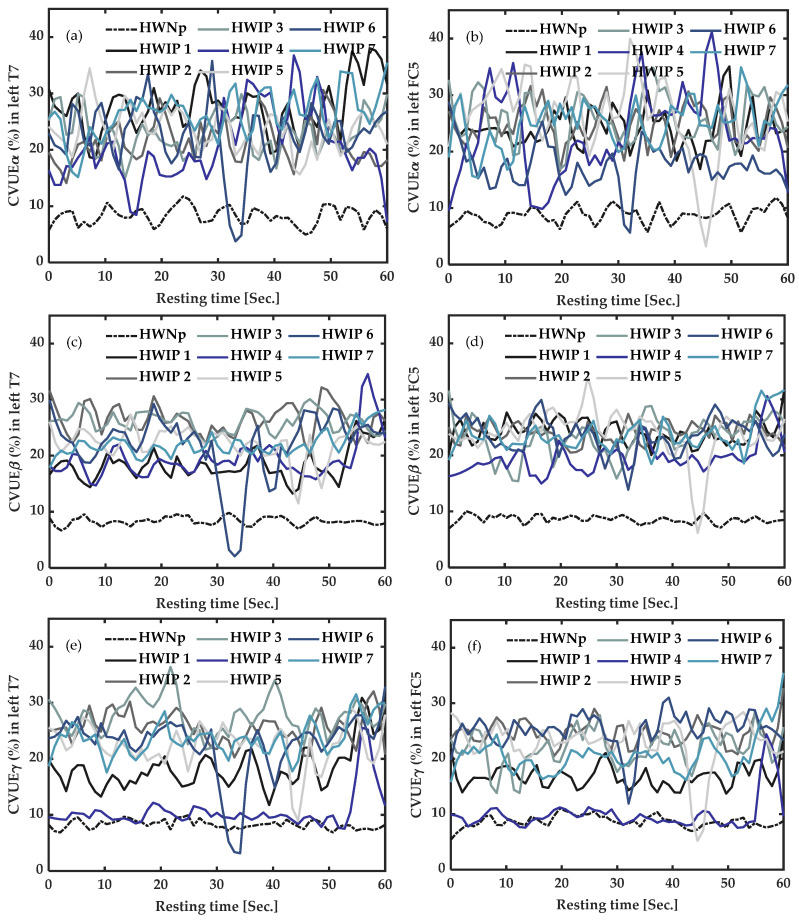
Baseline measures of the coefficient of variation of upper envelope (CVUE) in alpha (CVUE_α_), beta (CVUE_β_) and gamma (CVUE_γ_) at rest. (**a**)—CVUE_α_ from left temporal (T7) data, (**b**)—CVUE_α_ from left fronto-central (FC5) data, (**c**)—CVUE_β_ from left temporal (T7) data, (**d**)—CVUE_β_ from left fronto-central (FC5) data, (**e**)—CVUE_γ_ from left temporal (T7) data, (**f**)—CVUE_γ_ from left fronto-central (FC5) data. The black dotted line indicates the average of CVUE of healthy with “No pain” (HWNp). The colored lines represent CVUE of the seven healthy subjects with induced pain (HWIP_1-7_). The graphs were smoothed by a 5th-order of Savitzky–Golay filter. CVUE_β_ from FC5 data were significantly increased in all HWIP.

As shown in Figure 4, at rest, the CVUE of the seven participants with induced pain (HWIP) presented high values compared to their average with “No pain” (HWNp) represented by the black dotted line. The increase was more pronounced for CVUE_β_ from FC5 (*p* < 0.001) (Figure 4d), reflecting neuronal desynchronization (more disinhibition) of the motor cortex area.

### 3.4. EEG Active Measures: Coefficient of Variation of Upper Envelope from Alpha (CVUE_α_), Beta (CVUE_β_) and Gamma (CVUE_γ_) EEG Frequency Bands, during Task Execution

#### 3.4.1. In Participants with Chronic Fibromyalgia Pain (CFP) vs. Healthy with “No Pain” (HWNp) during Task Execution

**Figure 5:**
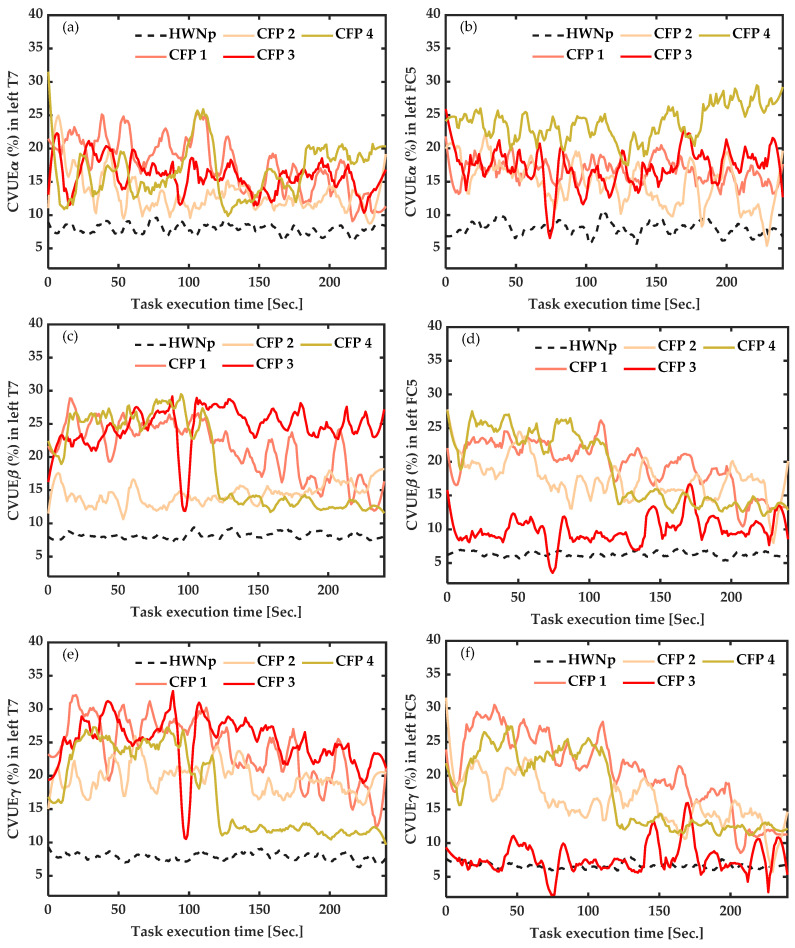
Active measures of the coefficient of variation of upper envelope (CVUE) in alpha (CVUE_α_), beta (CVUE_β_) and gamma (CVUE_γ_), during task execution. (**a**)—CVUE_α_ from left temporal (T7) data, (**b**)—CVUE_α_ from left fronto-central (FC5) data, (**c**)—CVUE_β_ from left temporal (T7) data, (**d**)—CVUE_β_ from left fronto-central (FC5) data, (**e**)—CVUE_γ_ from left temporal (T7) data, (**f**)—CVUE_γ_ from left fronto-central (FC5) data. The black dotted line indicates the average of CVUE of the healthy with “No pain” (HWNp). The colored lines represent CVUE of the four participants with chronic fibromyalgia pain (CFP_1-4_). The graphs were smoothed by a 15th-order of Savitzky–Golay filter. CVUE_β_ and CVUE_γ_ from T7 data were significantly increased in all CFP.

As shown in Figure 5, under task execution, the average CVUE of the seven healthy participants with “No pain” (HWNp) was less than 10%. This increase in the CVUE of CFP was more pronounced for CVUE_β_ from T7 (Figure 5c) and CVUE_γ_ from T7 (Figure 5e), suggesting neuronal desynchronization (more disinhibition) of the primary motor cortex area. 

#### 3.4.2. In Healthy Participants with Induced Pain (HWIP) vs. Healthy with “No Pain” (HWNp) during Task Execution

**Figure 6:**
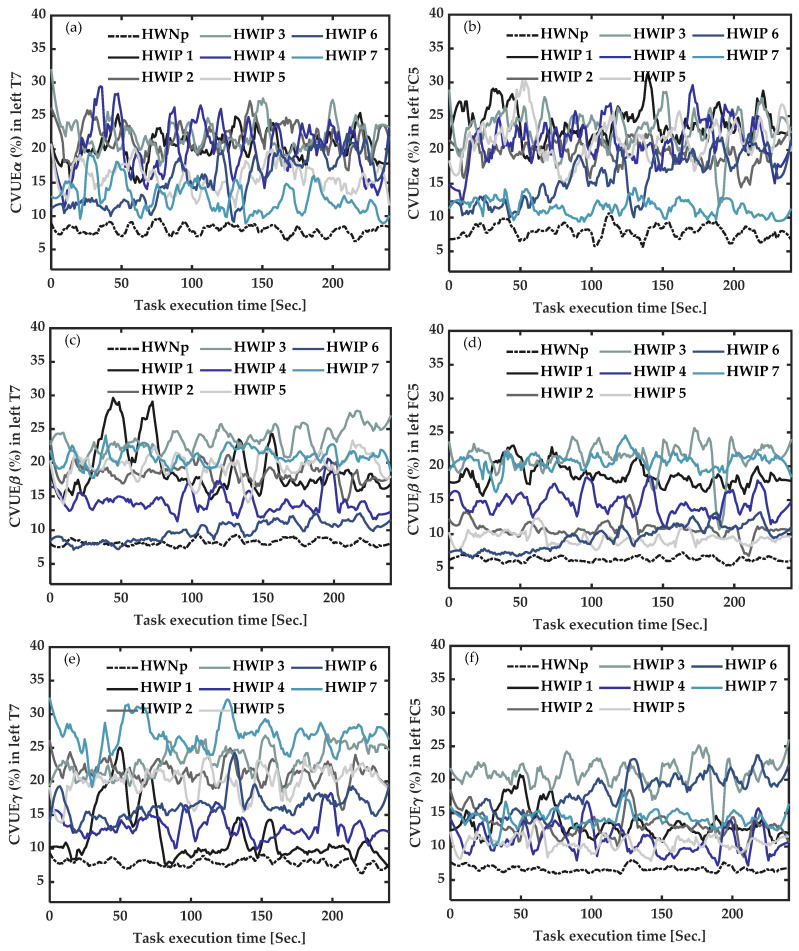
Active measures of the coefficient of variation of upper envelope (CVUE) in alpha (CVUE_α_), beta (CVUE_β_) and gamma (CVUE_γ_), during task execution. (**a**)—CVUE_α_ from left temporal (T7) data, (**b**)—CVUE_α_ from left fronto-central (FC5) data, (**c**)—CVUE_β_ from left temporal (T7) data, (**d**)—CVUE_β_ from left fronto-central (FC5) data, (**e**)—CVUE_γ_ from left temporal (T7) data, (**f**)—CVUE_γ_ from left fronto-central (FC5) data. The black dotted line indicates the average of CVUE of healthy with “No pain” (HWNp). The colored lines represent CVUE of the seven healthy with induced pain (HWIP_1-7_). The graphs were smoothed by a 15th-order of Savitzky–Golay filter. All CVUE (CVUE_α,_ CVUE_β,_ and CVUE_γ_) from left FC5 were higher in HWIP.

As illustrated in Figure 6, during task execution, CVUE of the seven participants with induced pain showed high values versus the average CVUE of the seven healthy participants with “No pain”. This difference was more pronounced for CVUE_β_ from FC5 (Figure 6d), indicating neuronal desynchronization (more disinhibition) of the motor cortex area.

In summary, we observed significantly high values of CVUE_β_ from FC5 at rest, in CFP participants and in HWIP (at rest and under task execution). Moreover, high values were also observed for CVUE_β_ and CVUE_γ_ from T7 during active measures (under task execution) in CFP. Subsequently, CVUE_β_ appears to be the most sensitive indicator for the identification of the presence of pain in an EEG signal. Therefore, CVUE_β_ from left FC5 was chosen for the subsequent comparisons and correlation analyses.

**Figure 7:**
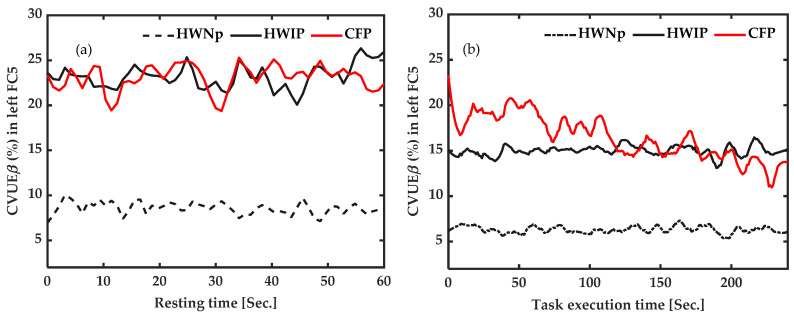
Baseline and active measures of the coefficient of variation of upper envelope (CVUE) in beta (CVUE_β_) from FC5 in the three groups. The black dotted line indicates the average of CVUE_β_ of healthy subjects with “No pain” (HWNp). The average of CVUE_β_ in (1) the seven participants with induced pain (HWIP) (black line) and (2) the four chronic pain (CFP) subjects (red line), at rest (**a**) and during task (**b**). CVUE_β_ from FC5 was significantly higher in participants both with chronic pain and experimental pain in comparison to healthy participants with “No pain”, at rest.

Figure 7 illustrates the average time-course of CVUE_β_ from left FC5 for the three groups, i.e., healthy participants with “No pain”—HWNp—(*n* = 7), participants with chronic pain—CFP—(*n* = 4) and healthy participants with induced pain—HWIP—(*n* = 7); both at baseline (at rest) and during active measures (under task execution). CVUE_β_ from FC5 was significantly higher in CFP (23.07%) and HWIP (23.2%) at rest (Figure 7a), versus in CFP (16.49%) and HWIP (14.99%) in active measures (Figure 7b). These results suggest that the identification of the presence of pain could be carried out from the data collected on the FC5 electrode, in beta frequency, with the participants at rest.

Finally, no significant correlation between CVUE_β_ and pain intensity or task performance was found.

## 4. Discussion

This proof-of-concept study aimed to explore the potential of developing objective pain identification based on the analysis of EEG signals. The main result is that the presence of pain is objectively identified by the increase (up to 20%) in the coefficient of variation of the upper envelope (CVUE) in the beta frequency band (12–30 Hz), i.e., CVUE_β_ captured at the left FC5 electrode, at rest. No significant correlation was found, suggesting a non-linear relationship between brain processes and clinical manifestations of a chronic fibromyalgia pain condition.

Our results are consistent with previous studies, as the increase in CVUE_β_ is indicative of greater cortical disinhibition [26,54,59,60,61,62]. Indeed, it is well known that nervous system plasticity is a fundamental property that provides the ability to adapt and incorporate genetic, developmental and environmental variations [63]. Therefore, counterbalancing mechanisms that maintain network stability is critically important. However, cortical disinhibition in the context of persistent pain may be interpreted as an alteration of the compensatory regulatory mechanisms of the neuronal system. Hence, the increase in CVUE_β_ can be considered as a neurophysiological signature of the activity of the global brain network in a chronic fibromyalgia pain condition.

To the best of our knowledge, very few studies have used CVUE to analyze EEG signal morphology in the context of pain. The originality of our approach is the focus on the detection of pain: pain or no pain? Several methods have previously been used to detect pain intensity, including the skin conductance approach [64], heart rate [65], heart rate variability [66] and blood pressure measures [67]. In the field of EEG studies, the perception of pain intensity has also often been studied. For example, Panavaranan and Wongsawat [68] conducted a study to detect acute thermal pain in humans using power spectral density (PSD)-based features as a pain index that are extracted from the EEG signals. They used a support vector machine (SVM) classifier to classify EEG signals into two different states: no pain (low PSD in alpha) and pain (high PSD in beta band), with an accuracy of 96.97%. Nir et al. [20] carried out the perception of tonic pain through a thermal contact-heat simulator. They used EEG signals for the characterization of peak alpha. Their results reported that increased peak alpha frequency values derived from EEG recordings of resting state, and noxious conditions were correlated with higher pain intensity. To determine the level of pain intensity in experimental conditions, Vatankhah et al. combined non-linear features, including approximate entropy, Lyapunov exponent, fractal dimension and energy ratios to constitute a pertinent feature vector. The participants verbally reported the perceived intensity rate of pain. All these selected features were computed to form a single vector, i.e., a pertinent feature vector [69]. Then, a hybrid adaptive network fuzzy inference system (ANFIS) and support vector machine (SVM) scheme were used to classify pain level intensity. Wager et al. [18] developed an fMRI-based measure that predicts pain intensity. They applied thermal stimuli in randomized sequences of varying intensity to the left forearm of each participant during fMRI scanning. Then, they used machine learning analyses to identify a pattern of fMRI activity across brain regions as a neurophysiological signature that was associated with heat-induced pain. The significant pattern showed sensitivity and specificity up to 94% in discriminating painful heat from non-painful warmth. 

In addition, in most pain studies using EEG [18,20,21,68], the intensity of pain was determined by considering the features in the frequency domain and in acute experimental pain conditions, and generally only at rest. The studies in the frequency domain use the Fast Fourier Transform (FFT) technique. Knowing that pain has a time-varying character and that the brain signal changes over time, the non-stationarity of the EEG signal remains a major challenge in its analysis. The FFT is only based on the hypothesis of the stationarity of the considered signals, and therefore does not account for time-varying characteristics, which justifies another approach, considering this challenge. Therefore, rather than making a frequency domain approach, we propose a time-frequency domain analysis that has the advantage of avoiding EEG signal distortion. Moreover, the envelope of the EEG signal has been shown to correlate with relevant aspects of the EEG signal morphology, which remains stable over time [54]; Thus, the extraction of the characteristics of the EEG signal from its envelope seems to be a relevant alternative [27]. This approach, based on the signal’s morphological stability, over time, shows great methodological robustness.

On the other hand, our results highlight the beta band (12–30 Hz) as the most sensitive frequency, in agreement with the previous studies showing this frequency band as most suitable for the study of pain conditions [41,70]. Some studies reported that women exhibit more pain intensity than men [71,72]. However, we did not find a difference in pain intensity between CFP (*n* = 4 women) and HWIP (*n* = 6 men), at rest. This is congruent with the literature [73]. Furthermore, no correlation was found between CVUE_β_ and pain intensity (at rest), in both groups (CFP/HWIP). Despite a much lower motor performance index than that of subjects with induced pain, no correlation was found between CVUE_β_ and motor performance in CFP, reinforcing the fact that motor response to chronic fibromyalgia pain is more complex and variable. However, our results seem to indicate a beneficial effect of the motor task on brain processes in the context of pain since we observed an average CVUE_β_ of around 16% during the task, versus 23% at rest in the chronic fibromyalgia pain condition, and 15% during the task versus 23% at rest in the acute experimental pain condition. This latter information projects a potential use of CVUE_β_ as a follow-up measure for task-oriented management of pain in rehabilitation.

Our idea was to improve the self-declaration of pain by a method that could be beneficial, especially for people who are unable to express their physical pain. Our results seem to highlight the optimal identification of the presence of pain when the participant is at rest, which would be very favorable in all pain conditions. However, some limitations need to be addressed. The sample size was small, and participants were not matched according to age, gender or pain duration. Klimesch reported that differences of amplitude in alpha EEG frequency were related to age [74]. Although other pain studies report that the beta band is the best one in painful conditions [75,76,77,78], in this proof-of-concept study, we also compared results across three frequency bands (alpha, beta and gamma); our results (Figure 1, Figure 2, Figure 3 and Figure 4) are in agreement with the literature on the relevance of the beta band. However, our best results were obtained in the beta, with no significant correlation with age and pain duration. Finally, we did not verify the effect of drugs on changes in CVUE. Even if J. B. Hargrove et al. [79] conducted a study on 85 participants living with fibromyalgia pain, they concluded that the use of drugs does not influence the emergence of specific EEG patterns. In all, since this proof-of-concept was to demonstrate the feasibility of the use of the CVUE method as to whether yes there is or no there is not the presence of pain, it would be advisable to perform future studies with larger samples to validate our results and remove any reasonable doubt about the effect of certain variables such as drug, age or gender.

## 5. Conclusions

This proof-of-concept study is a major step towards the objective identification of the presence of pain. In summary, (1) the Hilbert transform takes into account the complexity of the EEG signal, and (2) the envelope of the signal respects its morphology; it therefore remains constant over time despite the variation in the signal in the context of the very variable pain phenomenon. These are the two fundamental aspects of the methodological approach that we propose here and which make the analysis of brain activity appropriate and relevant in the context of pain. We have found that the coefficient of variation of the upper envelope (CVUE) in the beta frequency band (12–30 Hz), captured from EEG signals at the left FC5 electrode, could make it possible to objectively identify the presence of pain: CVUE_β_ up to 20% in chronic and experimental pain conditions at rest. This result has a strong implication in healthcare because it could allow the establishment of a specific objective diagnosis of the presence of pain, which could subsequently contribute to improved clinical decisions and the management of pain in various contexts. 

## Figures and Tables

**Table 1:** Participants’ characteristics.

	Chronic Fibromyalgia Pain Group (*n* = 4 Women)	Healthy Volunteers’ Group (*n* = 7)					
		Men (*n* = 6)		Woman (*n* = 1)		Mean	
Age (years)	42.75	28.67		45		36.83	
Pain duration (years)	19.5	-		-		-	
Pain intensity (NRS: 0–10)	4.75	3.83		5		4	
WPI (/19 body pain sides)	13.75	-		-		-	
Task Performance Index (/1)	0.02	with “No pain”	with induced pain	with “No pain”	with induced pain	with “No pain”	with induced pain
		0.59	0.63	0.39	0.39	0.56	0.59

## References

[1] Lynch M.E., Campbell F., Clark A.J., Dunbar M.J., Goldstein D.H., Peng P., Stinson J., Tupper H. (2008). A systematic review of the effect of waiting for treatment for chronic pain. Pain.

[2] Breivik H., Collett B., Ventafridda V., Cohen R., Gallacher D. (2006). Survey of chronic pain in Europe: Prevalence, impact on daily life, and treatment. Eur. J. Pain.

[3] Kennedy J., Roll J.M., Schraudner T., Murphy S., McPherson S. (2014). Prevalence of persistent pain in the US adult population: New data from the 2010 national health interview survey. J. Pain.

[4] Vos T., Abajobir A.A., Abate K.H., Abbafati C., Abbas K.M., Abd-Allah F., Abdulkader R.S., Abdulle A.M., Abebo T.A., Abera S.F. (2017). Global, regional, and national incidence, prevalence, and years lived with disability for 328 diseases and injuries for 195 countries, 1990–2016: A systematic analysis for the Global Burden of Disease Study 2016. Lancet.

[5] Turk D.C., Melzack R., Turk D.C., Melzack R. (2011). The Measurement of Pain and the Assessment of People Experiencing Pain. Handbook of Pain Assessment.

[6] Vos T., Flaxman A.D., Naghavi M., Lozano R., Michaud C., Ezzati M., Shibuya K., Salomon J.A., Abdalla S., Aboyans V. (2012). Years lived with disability (YLDs) for 1160 sequelae of 289 diseases and injuries 1990–2010: A systematic analysis for the Global Burden of Disease Study 2010. Lancet.

[7] Reid K.J., Harker J., Bala M.M., Truyers C., Kellen E., Bekkering G.E., Kleijnen J. (2011). Epidemiology of chronic non-cancer pain in Europe: Narrative review of prevalence, pain treatments and pain impact. Curr. Med. Res. Opin..

[8] Desmeules J.A., Cedraschi C., Rapiti E., Baumgartner E., Finckh A., Cohen P., Dayer P., Vischer T.L. (2003). Neurophysiologic evidence for a central sensitization in patients with fibromyalgia. Arthritis Rheumatol..

[9] Williams A.D., Gracely H.R. (2007). Biology and therapy of fibromyalgia. Functional magnetic resonance imaging findings in fibromyalgia. Arthritis Res. Ther..

[10] Shieh J.-S., Dai C.-Y., Wen Y.-R., Sun W.-Z. (2007). A novel fuzzy pain demand index derived from patient-controlled analgesia for postoperative pain. IEEE Trans. Biomed. Eng..

[11] Kamdar M.M. (2010). Principles of Analgesic Use in the Treatment of Acute Pain and Cancer Pain.

[12] Walsh T. (1984). Practical problems in pain measurements. Pain.

[13] Schnakers C., Zasler N.D. (2007). Pain assessment and management in disorders of consciousness. Curr. Opin. Neurol..

[14] Herr K., Coyne P.J., McCaffery M., Manworren R., Merkel S. (2011). Pain assessment in the patient unable to self-report: Position statement with clinical practice recommendations. Pain Manag. Nurs..

[15] Ploner M., May E.S. (2018). Electroencephalography and magnetoencephalography in pain research—Current state and future perspectives. Pain.

[16] Ploner M., Sorg C., Gross J. (2017). Brain rhythms of pain. Trends Cogn. Sci..

[17] Huang G., Xiao P., Hung Y., Iannetti G., Zhang Z., Hu L. (2013). A novel approach to predict subjective pain perception from single-trial laser-evoked potentials. Neuroimage.

[18] Wager T.D., Atlas L.Y., Lindquist M.A., Roy M., Woo C.-W., Kross E. (2013). An fMRI-based neurologic signature of physical pain. N. Engl. J. Med..

[19] Kumbhare D., Elzibak A.H., Noseworthy M.D. (2017). Evaluation of chronic pain using magnetic resonance (MR) neuroimaging approaches. Clin. J. Pain.

[20] Nir R.-R., Sinai A., Raz E., Sprecher E., Yarnitsky D. (2010). Pain assessment by continuous EEG: Association between subjective perception of tonic pain and peak frequency of alpha oscillations during stimulation and at rest. Brain Res..

[21] Nir R.-R., Sinai A., Moont R., Harari E., Yarnitsky D. (2012). Tonic pain and continuous EEG: Prediction of subjective pain perception by alpha-1 power during stimulation and at rest. Clin. Neurophysiol..

[22] Hu L., Iannetti G. (2019). Neural indicators of perceptual variability of pain across species. Proc. Natl. Acad. Sci. USA.

[23] May E.S., Nickel M.M., Dinh S.T., Tiemann L., Heitmann H., Voth I., Tölle T.R., Gross J., Ploner M. (2019). Prefrontal gamma oscillations reflect ongoing pain intensity in chronic back pain patients. Hum. Brain Mapp..

[24] Wei C.-S., Keller C.J., Li J., Lin Y.-P., Nakanishi M., Wagner J., Wu W., Zhang Y., Jung T.-P. (2021). Editorial: Inter-and Intra-subject Variability in Brain Imaging and Decoding. Front. Comput. Neurosci..

[25] Cole S.R., Voytek B. (2017). Brain oscillations and the importance of waveform shape. Trends Cogn. Sci..

[26] Díaz J., Razeto-Barry P., Letelier J.-C., Caprio J., Bacigalupo J. (2007). Amplitude modulation patterns of local field potentials reveal asynchronous neuronal populations. J. Neurosci..

[27] Díaz J., Arancibia J.M., Bassi A., Vivaldi E.A. (2014). Envelope analysis of the airflow signal to improve polysomnographic assessment of sleep disordered breathing. Sleep.

[28] Romaine J., Martín M.P., Ortiz J.S., Crespo J.M. (2021). EEG—Single-Channel Envelope Synchronisation and Classification for Seizure Detection and Prediction. Brain Sci..

[29] Katz J.S. Scale-Independent Measures: Theory and Practice. Proceedings of the 17th International Conference on Science and Technology Indicators.

[30] Wolfe F., Smythe H.A., Yunus M.B., Bennett R.M., Bombardier C., Goldenberg D.L., Tugwell P., Campbell S.M., Abeles M., Clark P. (1990). The American College of Rheumatology 1990 criteria for the classification of fibromyalgia. Arthritis Rheumatol..

[31] Edlin J.M., Leppanen M., Fain R.J., Hackländer R.P., Hanaver-Torrez S.D., Lyle K.B. (2015). On the use (and misuse?) of the Edinburgh Handedness Inventory. Brain Cogn..

[32] Oldfield R.C. (1971). The assessment and analysis of handedness: The Edinburgh inventory. Neuropsychologia.

[33] Karcioglu O., Topacoglu H., Dikme O., Dikme O. (2018). A systematic review of the pain scales in adults: Which to use?. Am. J. Emerg. Med..

[34] Wolfe F., Clauw D.J., Fitzcharles M.-A., Goldenberg D.L., Katz R.S., Mease P., Russell A.S., Russell I.J., Winfield J.B., Yunus M.B. (2010). The American College of Rheumatology preliminary diagnostic criteria for fibromyalgia and measurement of symptom severity. Arthritis Care Res. Hoboken.

[35] Bouffard J., Bouyer L.J., Roy J.-S., Mercier C. (2016). Pain induced during both the acquisition and retention phases of locomotor adaptation does not interfere with improvements in motor performance. Neural Plast..

[36] Lomond K.V., Côté J.N. (2010). Movement timing and reach to reach variability during a repetitive reaching task in persons with chronic neck/shoulder pain and healthy subjects. Exp. Brain Res..

[37] Flake G.W., Baum E.B. (2002). Rush Hour is PSPACE-complete, or “Why you should generously tip parking lot attendants”. Theor. Comput. Sci..

[38] Athif M., Ren H. (2019). WaveCSP: A robust motor imagery classifier for consumer EEG devices. Australas. Phys. Eng. Sci. Med..

[39] Burns E., Chipchase L., Schabrun S. (2016). Primary sensory and motor cortex function in response to acute muscle pain: A systematic review and meta-analysis. Eur. J. Pain.

[40] Bouffard J., Bouyer L.J., Roy J.-S., Mercier C. (2014). Tonic pain experienced during locomotor training impairs retention despite normal performance during acquisition. J. Neurosci..

[41] Segning C., Ezzaidi H., da Silva R., Ngomo S. (2021). A Neurophysiological Pattern as a Precursor of Work-Related Musculoskeletal Disorders Using EEG Combined with EMG. Int. J. Environ. Res. Public Health.

[42] Power J.D., Lynch C.J., Dubin M.J., Silver B.M., Martin A., Jones R.M. (2020). Characteristics of respiratory measures in young adults scanned at rest, including systematic changes and “missed” deep breaths. Neuroimage.

[43] Liberati G., Klöcker A., Algoet M., Mulders D., Safronova M.M., Santos S.F., Vaz J.-G.R., Raftopoulos C., Mouraux A. (2018). Gamma-band oscillations preferential for nociception can be recorded in the human insula. Cereb. Cortex.

[44] Shao S., Shen K., Yu K., Wilder-Smith E., Li X. (2012). Frequency-domain EEG source analysis for acute tonic cold pain perception. Clin. Neurophysiol..

[45] Aboalayon K.A.I., Almuhammadi W.S., Faezipour M. A Comparison of Different Machine Learning Algorithms Using Single Channel EEG Signal for Classifying Human Sleep Stages. Proceedings of the 2015 Long Island Systems, Applications and Technology.

[46] Zhang X., Yao L., Zhang D., Wang X., Sheng Q.Z., Gu T. Multi-person brain activity recognition via comprehensive EEG signal analysis. Proceedings of the 14th EAI International Conference on Mobile and Ubiquitous Systems: Computing, Networking and Services.

[47] Zhao Y., Cen Y. (2013). Data Mining Applications with R.

[48] Cohen M.X. (2017). MATLAB for Brain and Cognitive Scientists.

[49] Yousif E.S., Abdulbaqi A.S., Hameed A.Z., Al-din S. (2020). Electroencephalogram Signals Classification Based on Feature Normalization. Dans IOP Conf. Ser. Mater. Sci. Eng..

[50] Boashash B. (2015). Time-Frequency and Instantaneous Frequency Concepts. Dans Time-Frequency Signal Analysis and Processing: A Comprehensive Reference.

[51] Sanei S., Chambers J.A. (2013). Fundamentals of EEG Signal Processing. Dans EEG Signal Processing.

[52] Park I., Díaz J., Matsumoto S., Iwayama K., Nabekura Y., Ogata H., Kayaba M., Aoyagi A., Yajima K., Satoh M. (2021). Exercise improves the quality of slow-wave sleep by increasing slow-wave stability. Sci. Rep..

[53] Zamm A., Debener S., Bauer A.-K.R., Bleichner M.G., Demos A.P., Palmer C. (2018). Amplitude envelope correlations measure synchronous cortical oscillations in performing musicians. Ann. N. Y. Acad. Sci..

[54] Díaz J., Bassi A., Coolen A., Vivaldi E.A., Letelier J.-C. (2018). Envelope analysis links oscillatory and arrhythmic EEG activities to two types of neuronal synchronization. Neuroimage.

[55] Charier D.J., Zantour D., Pichot V., Chouchou F., Barthelemy J.-C.M., Roche F., Molliex S.B. (2017). Assessing pain using the variation coefficient of pupillary diameter. J. Pain.

[56] Mani S., Sharma S., Singh D.K. (2019). Concurrent validity and reliability of telerehabilitation-based physiotherapy assessment of cervical spine in adults with non-specific neck pain. J. Telemed. Telecare.

[57] Maquet D., Croisier J.-L., Demoulin C., Crielaard J.-M. (2004). Pressure pain thresholds of tender point sites in patients with fibromyalgia and in healthy controls. Eur. J. Pain.

[58] Maris E., Oostenveld R. (2007). Nonparametric statistical testing of EEG-and MEG-data. J. Neurosci. Methods.

[59] Volz M.S., Medeiros L.F., Tarragô M.D.G., Vidor L.P., Dall’agnol L., Deitos A., Brietzke A., Rozisky J.R., Rispolli B., Torres I.L. (2013). The Relationship between Cortical Excitability and Pain Catastrophizing in Myofascial Pain. J. Pain.

[60] Vidor L.P., Torres I.L., Medeiros L.F., Dussán-Sarria J.A., Dall’Agnol L., Deitos A., Brietzke A., Laste G., Rozisky J.R., Fregni F. (2014). Association of anxiety with intracortical inhibition and descending pain modulation in chronic myofascial pain syndrome. BMC Neurosci..

[61] Caumo W., Deitos A., Carvalho S., Leite J., Carvalho F., Dussan-Sarria J., Tarragó M.D.G.L., Souza A., Torres I.L., Fregni F. (2016). Motor cortex excitability and BDNF levels in chronic musculoskeletal pain according to structural pathology. Front. Hum. Neurosci..

[62] Botelho M.L., Morales-Quezada L., Rozisky R.J., Brietzke P.A., Torres L.I., Deitos A., Caumo W. (2016). A framework for understanding the relationship between descending pain modulation, motor corticospinal, and neuroplasticity regulation systems in chronic myofascial pain. Front. Hum. Neurosci..

[63] Yin J., Yuan Q. (2015). Structural homeostasis in the nervous system: A balancing act for wiring plasticity and stability. Front. Cell. Neurosci..

[64] Boucsein W. (2012). Electrodermal Activity.

[65] Terkelsen A.J., Mølgaard H., Hansen J., Andersen O.K., Jensen T.S. (2005). Acute pain increases heart rate: Differential mechanisms during rest and mental stress. Auton. Neurosci..

[66] Appelhans B.M., Luecken L.J. (2008). Heart rate variability and pain: Associations of two interrelated homeostatic processes. Biol. Psychol..

[67] Saccò M., Meschi M., Regolisti G., Detrenis S., Bianchi L., Bertorelli M., Pioli S., Magnano A., Spagnoli F., Giuri P.G. (2013). The relationship between blood pressure and pain. J. Clin. Hypertens..

[68] Panavaranan P., Wongsawat Y. EEG-Based Pain Estimation via Fuzzy Logic and Polynomial Kernel Support Vector Machine. Proceedings of the 6th 2013 Biomedical Engineering International Conference.

[69] Vatankhah M., Asadpour V., Fazel-Rezai R. (2013). Perceptual pain classification using ANFIS adapted RBF kernel support vector machine for therapeutic usage. Appl. Soft Comput..

[70] Alazrai R., Momani M., Abu Khudair H., Daoud M.I. (2019). EEG-based tonic cold pain recognition system using wavelet transform. Neural Comput. Appl..

[71] Frot M., Feine J.S., Bushnell M.C. (2004). Sex differences in pain perception and anxiety. A psychophysical study with topical capsaicin. Pain.

[72] Ramírez-Maestre C., Esteve R. (2014). The role of sex/gender in the experience of pain: Resilience, fear, and acceptance as central variables in the adjustment of men and women with chronic pain. J. Pain.

[73] Nickel M., May E.S., Tiemann L., Schmidt P., Postorino M., Dinh S.T., Gross J., Ploner M. (2017). Brain oscillations differentially encode noxious stimulus intensity and pain intensity. Neuroimage.

[74] Klimesch W. (1999). EEG alpha and theta oscillations reflect cognitive and memory performance: A review and analysis. Brain Res. Rev..

[75] Arendsen L.J., Guggenberger R., Zimmer M., Weigl T., Gharabaghi A. (2021). Peripheral electrical stimulation modulates cortical beta-band activity. Front. Neurosci..

[76] Hsiao J.F., Chen T.W., Liu Y.H., Wang F.Y., Chen P.S., Lai L.K., Hope Pan L., Coppola G., Wang S.J. (2021). Migraine chronification is associated with beta-band connectivity within the pain-related cortical regions: A magnetoencephalographic study. Pain.

[77] Kim J.A., Bosma R.L., Hemington K.S., Rogachov A., Osborne N.R., Cheng J.C., Oh J., Crawley A.P., Dunkley B.T., Davis K.D. (2019). Neuropathic pain and pain interference are linked to alpha-band slowing and reduced beta-band magnetoencephalography activity within the dynamic pain connectome in patients with multiple sclerosis. Pain.

[78] Wei M., Liao Y., Liu J., Li L., Huang G., Huang J., Li D., Xiao L., Zhang Z. (2022). EEG beta-band spectral entropy can predict the effect of drug treatment on pain in patients with herpes zoster. J. Clin. Neurophysiol..

[79] Hargrove J.B., Bennett R., Simons D.G., Smith S.J., Nagpal S., Deering D.E. (2010). Quantitative electroencephalographic abnormalities in fibromyalgia patients. Clin. EEG Neurosci..

